# Effect of Calcifications on Breast Ultrasound Shear Wave Elastography: An Investigational Study

**DOI:** 10.1371/journal.pone.0137898

**Published:** 2015-09-14

**Authors:** Adriana Gregory, Mohammad Mehrmohammadi, Max Denis, Mahdi Bayat, Daniela L. Stan, Mostafa Fatemi, Azra Alizad

**Affiliations:** 1 Department of Physiology and Biomedical Engineering, Mayo Clinic College of Medicine, Rochester, Minnesota, United States of America; 2 Department of Internal Medicine, Mayo Clinic College of Medicine, Rochester, Minnesota, United States of America; Universite de Nantes, FRANCE

## Abstract

**Purpose:**

To investigate the effects of macrocalcifications and clustered microcalcifications associated with benign breast masses on shear wave elastography (SWE).

**Methods:**

SuperSonic Imagine (SSI) and comb-push ultrasound shear elastography (CUSE) were performed on three sets of phantoms to investigate how calcifications of different sizes and distributions influence measured elasticity. To demonstrate the effect *in vivo*, three female patients with benign breast masses associated with mammographically-identified calcifications were evaluated by CUSE.

**Results:**

Apparent maximum elasticity (E_max_) estimates resulting from individual macrocalcifications (with diameters of 2mm, 3mm, 5mm, 6mm, 9mm, 11mm, and 15mm) showed values over 50 kPa for all cases, which represents more than 100% increase over background (~21kPa). We considered a 2cm-diameter circular region of interest for all phantom experiments. Mean elasticity (E_mean_) values varied from 26 kPa to 73 kPa, depending on the macrocalcification size. Highly dense clusters of microcalcifications showed higher E_max_ values than clusters of microcalcification with low concentrations, but the difference in E_mean_ values was not significant.

**Conclusions:**

Our results demonstrate that the presence of large isolated macrocalcifications and highly concentrated clusters of microcalcifications can introduce areas with apparent high elasticity in SWE. Considering that benign breast masses normally have significantly lower elasticity values than malignant tumors, such areas with high elasticity appearing due to presence of calcification in benign breast masses may lead to misdiagnosis.

## Introduction

Breast calcifications are mineral deposits that can develop due to various causes. Oftentimes, calcifications are present in both benign and malignant breast masses [1, 2]. Several studies have shown that calcifications in benign masses are mostly composed of calcium oxalate, while those of malignant masses are composed of calcium hydroxyapatite [3–7]. Moreover, size and distribution of breast calcifications differ among benign and malignant breast masses. Benign calcifications can appear as diffused or clustered patterns, and can be very small in size (microcalcifications) or large (macrocalcifications). Malignant calcifications usually form linear or segmental patterns, and are typically more dispersed and pleomorphic [1, 2, 8, 9].

Currently, mammography and ultrasonography are the most commonly used imaging tools for breast cancer detection. Compared to ultrasonography, mammography has a higher sensitivity of detecting calcifications. However, due to the relatively low sensitivity (67.8%) and specificity (75%) of mammography in cancer screening [10], new methods are emerging to improve these statistical measures. One technique is vibro-acoustography (VA), an imaging modality that is sensitive to the dynamic characteristics of tissue [11]. The *ex vivo* and *in vivo* studies of VA for breast lesion detection including macro- and microcalcifications have shown promising results [12–19]. Another emerging technique is elasticity imaging, including quasi-static elastography and shear wave elastography (SWE). The quasi-static techniques are mostly qualitative [20, 21], however, some display the modulus up to a multiplicative parameter, and are therefore quantitative in that sense. On the other hand, shear wave techniques are quantitative and use acoustic radiation force of ultrasound to generate shear waves in tissue. With shear wave elasticity imaging techniques, tissue elasticity can be calculated from measured shear wave speed [22–25]. Shear-wave-based elastography provides palpation-like information such as tissue stiffness. It is well known that malignant masses are usually stiffer than benign masses [26–29], for this reason, different SWE methods have been used to differentiate between benign and malignant masses in different organs. SuperSonic Imagine (SSI) [22, 30] and shear wave imaging using Acoustic Radiation Force Impulse (ARFI) [31, 32] are two of the most well-known SWE techniques. The advantages of these techniques in the differentiation of breast masses have been well studied [31, 33–39], but few studies have investigated the effect of breast calcifications on SWE [40–42].

When tissue is characterized by its physical properties like elasticity, it is important to consider what factors could affect the tissue stiffness and potentially lead to false positive or false negative results. Breast calcifications have a very different elastic behavior than normal or abnormal breast tissue and have a high incidence in mammograms (up to 80%) [43]. The elastic modulus (Young’s Modulus) of calcium oxalate (24.5GPa) and calcium hydroxyapatite (40-117GPa) [44, 45] is extremely large compared to the mean elasticity values (E_mean_) of benign masses (10-80kPa) and malignant masses (30-180kPa) [46–52]. It is expected that the corresponding shear wave speeds (if detectable at all) are well beyond the highest ultrasound imaging frame rate currently available for the SWE. The imaging and tracking artifacts, on the contrary, can give rise to erroneous elasticity readings on location of calcification which can be within the dynamic range of SWE techniques.

In this paper, we experimentally quantify how much the stiffness reading rises in the presence of calcifications on SWE. We have focused on the characteristics of benign calcifications and examined the effects of microcalcifications and macrocalcifications using comb-push ultrasound shear elastography (CUSE) and SuperSonic Imagine (SSI) techniques. The results of phantom studies of calcifications and *in vivo* CUSE evaluations of women with benign breast masses with calcifications are presented.

## Methods and Materials

### CUSE Imaging Method and System

**Fig 1:**
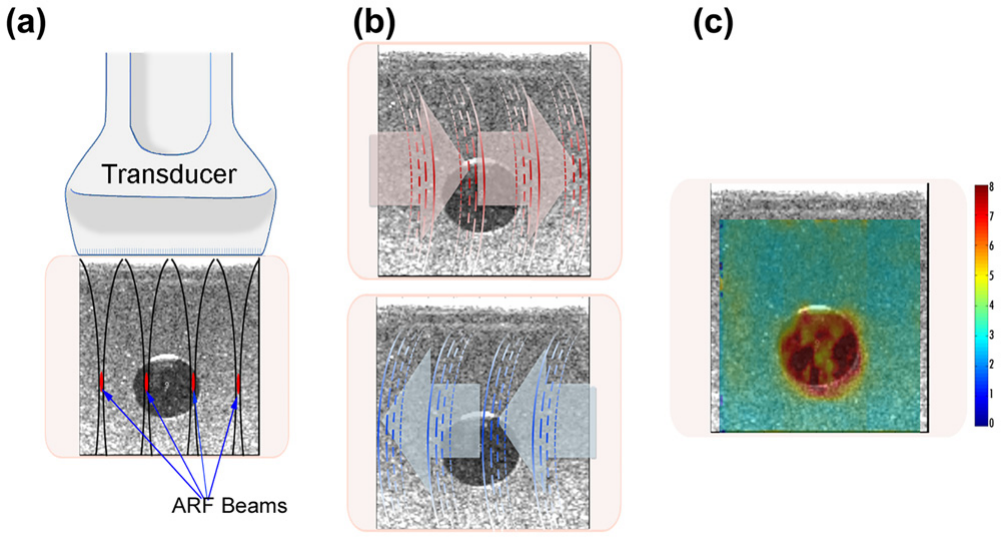
CUSE method. (a) Excitation of the medium by using four equidistant acoustic radiation force beams. (b) Detection of LR (*red arrows*) and RL (*blue arrows*) shear wave propagations. (c) Reconstruction of a 2D shear wave map from the calculated shear wave propagation speed.

CUSE is a fast ultrasound-based quantitative and two-dimensional shear wave elasticity imaging technique in which multiple laterally distributed acoustic radiation force beams are used to simultaneously excite the tissue and induce shear waves [53]. CUSE with four focused push beams is one of the shear wave imaging methods for this study (Fig 1a). Local shear wave speed was measured at each pixel by averaging the speed of left-to-right (LR) and right-to-left (RL) waves (Fig 1b). The calculated shear wave speed values were utilized to reconstruct a color-coded shear wave speed map (Fig 1c) [54].

For this study, we used CUSE that was implemented on a Verasonics V1 system (Verasonics Inc, Kirkland, WA, USA) equipped with a linear array transducer L7-4 (Philips Healthcare, Andover, MA), as described in Mehrmohammadi et. al [26]. B-mode image and in-phase/quadrature (IQ) data were acquired. The IQ data was processed by a Matlab-based (Mathworks Inc., MA) graphical user interface to reconstruct a shear wave speed map. A 5×5-pixel mean filter was applied to smooth the shear wave map. The B-mode image was overlaid with the shear wave speed map to obtain the final image. No denoising or frame averaging was performed.

### SSI System

**Fig 2:**
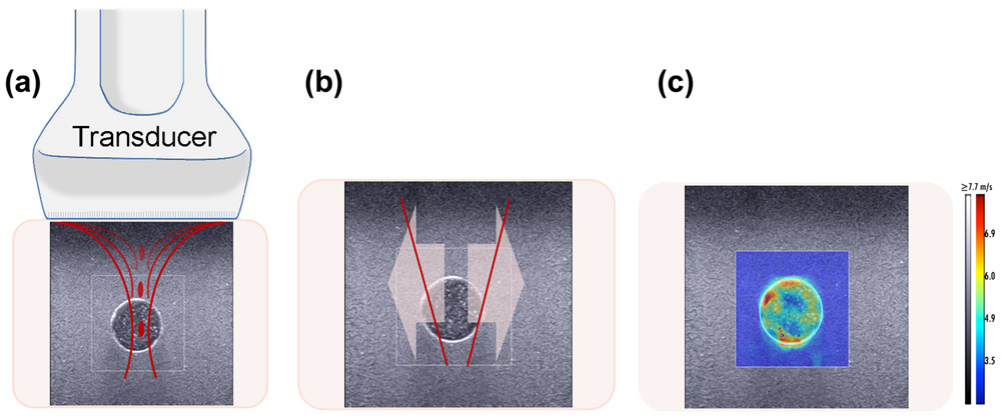
SSI method. (a) Excitation of medium by generating a sequence of acoustic radiation force points parallel to the beam axis. (b) Propagation of the two shear waves in opposite directions. (c) Reconstruction of a 2D elasticity map from measured local displacement.

SSI performed by an AixPlorer (SuperSonic Imagine, Aix en Provence, France) is a real-time 2D-SWE ultrasound-based shear imaging technique that provides elasticity information. This method excites tissue by generating acoustic radiation force at different focal points along the beam axis (Fig 2a) at a supersonic speed creating two shear waves that propagate in opposite directions (Fig 2b). By measuring and tracking the local displacements produced by the shear wave propagation, the system reconstructs an elasticity map (Fig 2c) [22].

The probe used for this study was a linear array transducer with a 4–15 MHz bandwidth (SuperLinear SL15-4, Aix en Provence, France). The color scale for the SWE images was fixed to the default given by the Aixplorer system (≥7.7m/s). SWE imaging was set to penetration mode. B-mode and elasticity images were collected in DICOM format and analyzed with the image processing software OsiriX (Pixmeo SARL, Geneva, Switzerland).

### Tissue Mimicking Phantom Studies

Three sets of experiments were performed on tissue mimicking phantoms to assess the effects of calcifications on SWE.

#### Experiment 1—Study on the effect of calcification size

**Fig 3:**
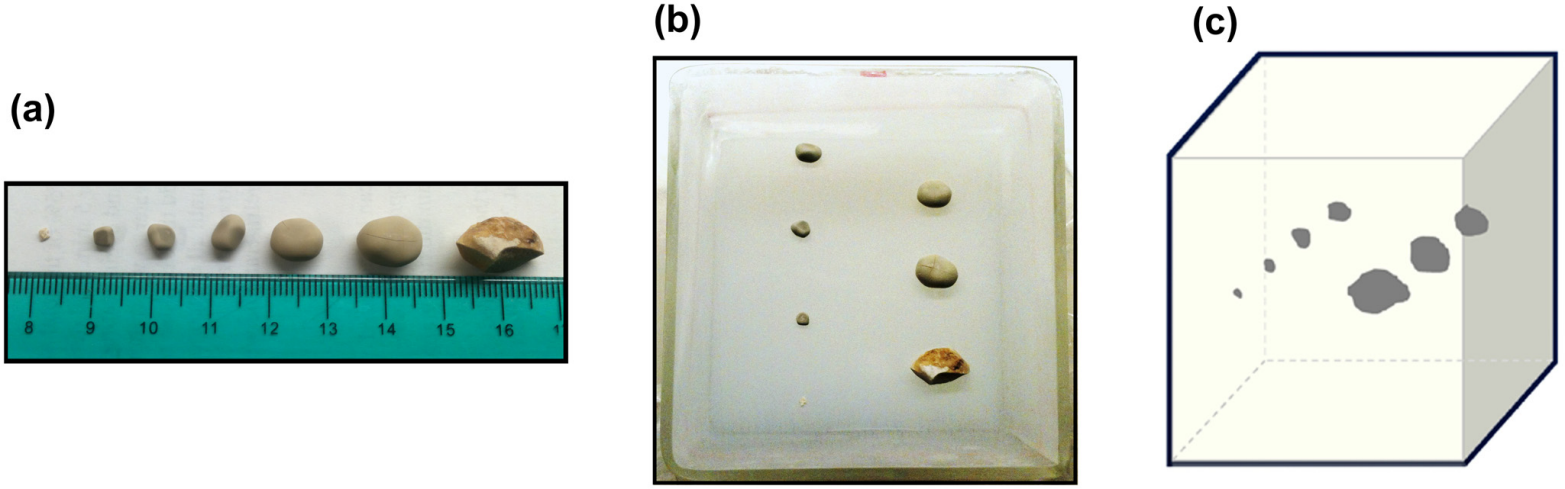
Experiment 1—Macrocalcifications phantom. (a) Kidney Stones used for the experiment. (b) Kidney stones placed on the gelatin phantom. (c) Diagram of the kidney stones inside the phantom.

The first experiment involved seven calcified inclusions within a tissue mimicking gelatin background. The calcified inclusions were made out of kidney stones (KS). The chemical composition of the kidney stones was calcium oxalate (CaC_2_O_4_) [55–59], which is similar to the composition of benign calcifications found in breast tissue [5]. The purpose of the phantom was to investigate the effect of macrocalcifications with different sizes on shear wave elasticity estimates. We selected seven kidney stones with diameters of 2mm, 3mm, 5mm, 6mm, 9mm, 11mm, and 15mm, to represent coarse or popcorn-like macrocalcifications. The phantom was made by mixing 75% v/v distilled water, 9% v/v gelatin from porcine skin (300 bloom, Sigma Aldrich, MO, US), 10% v/v Glycerol (Sigma Aldrich, MO, US) and 0.8% wt/wt Laponite (Sigma Aldrich, MO, US). Cellulose particles of 20μm and 50μm (Sigma Aldrich, MO, US) were added to the background. A first layer of gelatin mix was poured in a 729 cm^3^ cubic mold, and was covered with plastic membrane to set at room temperature. After 45 minutes, the seven kidney stones were placed on the phantom within a 9×9 cm area, then a second layer of gelatin mix was poured over them (Fig 3).

Once the second layer was set, the phantom was removed from the mold and placed on the base of a support stand. The probe was fixed on top of the phantom held by an extension clamp to avoid movement and pre-compression effects on shear wave imaging (SWI). Conventional B-mode was performed to localize the seven inclusions. SSI elasticity imaging was conducted first followed by CUSE. Each inclusion was imaged separately.

#### Experiment 2—Study on the effect of calcifications distribution

The second experiment involved three gelatin phantoms to evaluate the effect of calcifications with different distributions on SWE.

**Fig 4:**
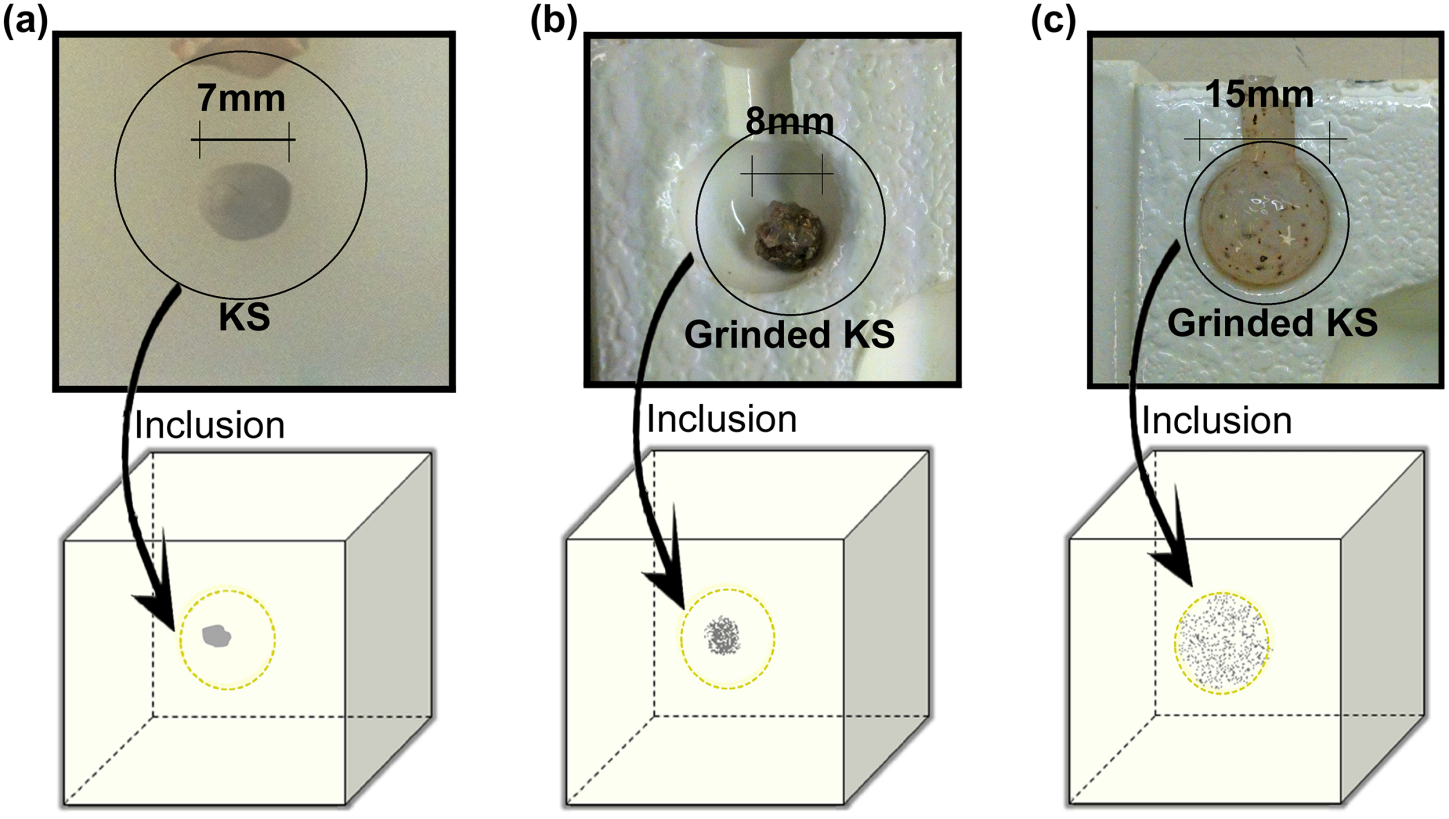
Experiment 2. (a) Inclusion with 7mm intact kidney stone. (b) Inclusion with clustered distribution of ground kidney stone. (c) Inclusion with regional scattered distribution of ground kidney stone.

The first phantom contained a 200mg intact kidney stone inclusion to simulate a very concentrated dystrophic, coarse or popcorn-like macrocalcification (Fig 4a). The second phantom inclusion was composed of 200mg ground kidney stone mixed with 0.2ml of tissue mimicking gelatin, resembling a concentrated cluster of round and punctate microcalcifications (Fig 4b). The third phantom inclusion was made by mixing 200mg of ground kidney stone with 1.77ml of tissue mimicking gelatin to resemble a diffused cluster of microcalcifications, similar to regional scattered microcalcifications (Fig 4c). Each inclusion was placed between two layers of gelatin background in 3 different 7×7×6cm cubic molds. The gelatin background recipe and imaging setup for the three phantoms was similar to those described for the first experiment.

#### Experiment 3 –Study of the apparent increase in shear wave speed due to presence of calcifications

Results obtained from the first experiment showed high shear wave speed regions below the larger kidney stones. The aim of this experiment is to determine the cause of such increase in shear wave speed. The fact that elevated speed appears asymmetrically, i.e., mainly below the calcification, suggest that this artifact could be caused by erroneous tracking of the shear waves. For this reason, this experiment was designed to simultaneously detect the shear waves at opposite sides of the phantom.

**Fig 5:**
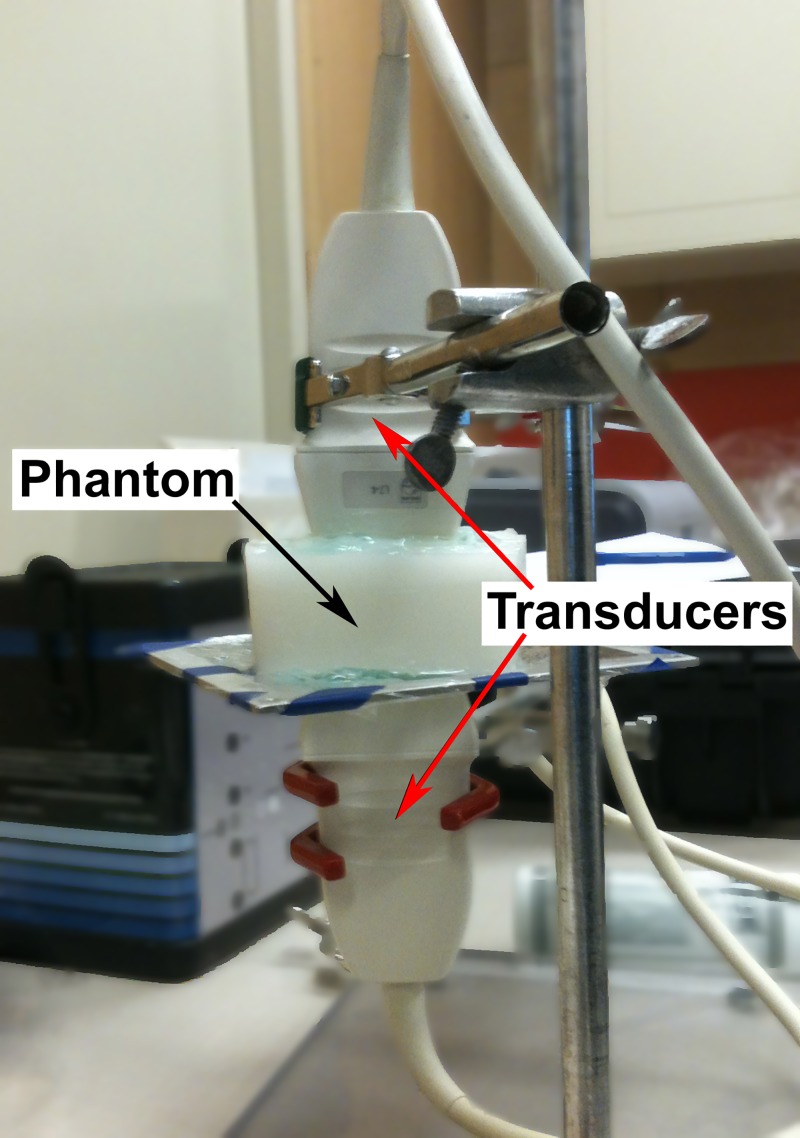
Experiment 3 –Imaging of a large kidney stone with two opposite transducers.

Two Verasonics systems with two L7-4 linear transducers were used for the experiment; each system had the same CUSE program. The phantom for the test consisted in a 9×9×4.5cm tissue mimicking gelatin background with an 11 mm kidney stone inclusion. The gelatin recipe was the same as for the previous experiments. The two L7-4 transducers were coaxially aligned facing each other with a gap (about the height of the phantom) in between and held by clamps on a support stand. The phantom was then placed between the probes on a grid attached to the support stand (Fig 5).

The first CUSE system had the transducer placed on top of the phantom. The second CUSE system with the transducer on the opposite side of the phantom was modified by eliminating the transmission of push beams, but it was still able to track the shear waves produce by the top transducer. The two systems were synchronized to allow simultaneous SWE imaging at the top and bottom of the phantom.

### *In vivo* Human Study

A total of three female patients with suspicious breast masses and mammographically identified calcifications were selected for this investigation. This study was approved by the Mayo Clinic Institutional Review Board (IRB). Written signed informed consents with permission for publication, approved by Mayo Clinic IRB, were obtained from enrolled patients. Each patient had received a clinical ultrasound and mammography prior to participating in the study. SWE evaluations were performed prior to biopsy.

Each patient was first evaluated by an experienced sonographer with conventional B-mode ultrasound to identify the breast mass. The mass area was marked on the image by freehand drawing to identify the ROI. Then, the probe was fixed in place by a lockable articulated arm. Thereafter, CUSE was conducted. In order to reduce breathing motion artifact, the patient was asked to hold her breath for each ultrasound and CUSE acquisition.

### Elasticity Measurements

To calculate the stiffness values of a specific location, a circular region of interest (ROI) with a diameter of 20mm (simulating a 20mm breast lesion) was selected across all the phantom samples. The ROI selection was operator dependent. To address the issue of variability, we took three shear wave acquisitions and for each acquisition we tried to position the calcified area in the middle of the 20mm ROI. Each measurement was independent of other measurements. For in vivo studies, the ROI was drawn following the boundaries of the lesion.

Minimum, maximum, mean, and standard deviation values of the shear wave speeds were obtained from CUSE images. To translate the shear wave speed into elasticity (Young’s Modulus) the following expression was used:

$$E=3\rho c^{2}$$ (1)

where *ρ* = 1000*Kg*/*m* ^3^ represents the tissue density and *c* is the shear wave speed. The above expression assumes that soft tissue is a linear, isotropic, incompressible and elastic material. In the case of SSI machine, the minimum, maximum, mean and standard deviation elasticity values (Young’s Modulus) were obtained after selecting the ROI with the SuperSonic Imagine’s QBox tool available with the OsiriX image processing software.

To further quantify the effects of calcifications, a relative error was defined as,

$$\text{Relative error(\%)}=\left(\frac{\left|E_{ROI}-E_{BG}\right|}{E_{BG}}\right)\times 100$$ (2)

where *E*_*ROI*_ and *E*_*BG*_ are the mean elasticity of the ROI and background, respectively. The relative error indicates the changes of the elasticity in the ROI relative to the elasticity of the background.

## Results

### Tissue mimicking phantom study results

#### Experiment 1—Study on the effect of calcification size

**Fig 6:**
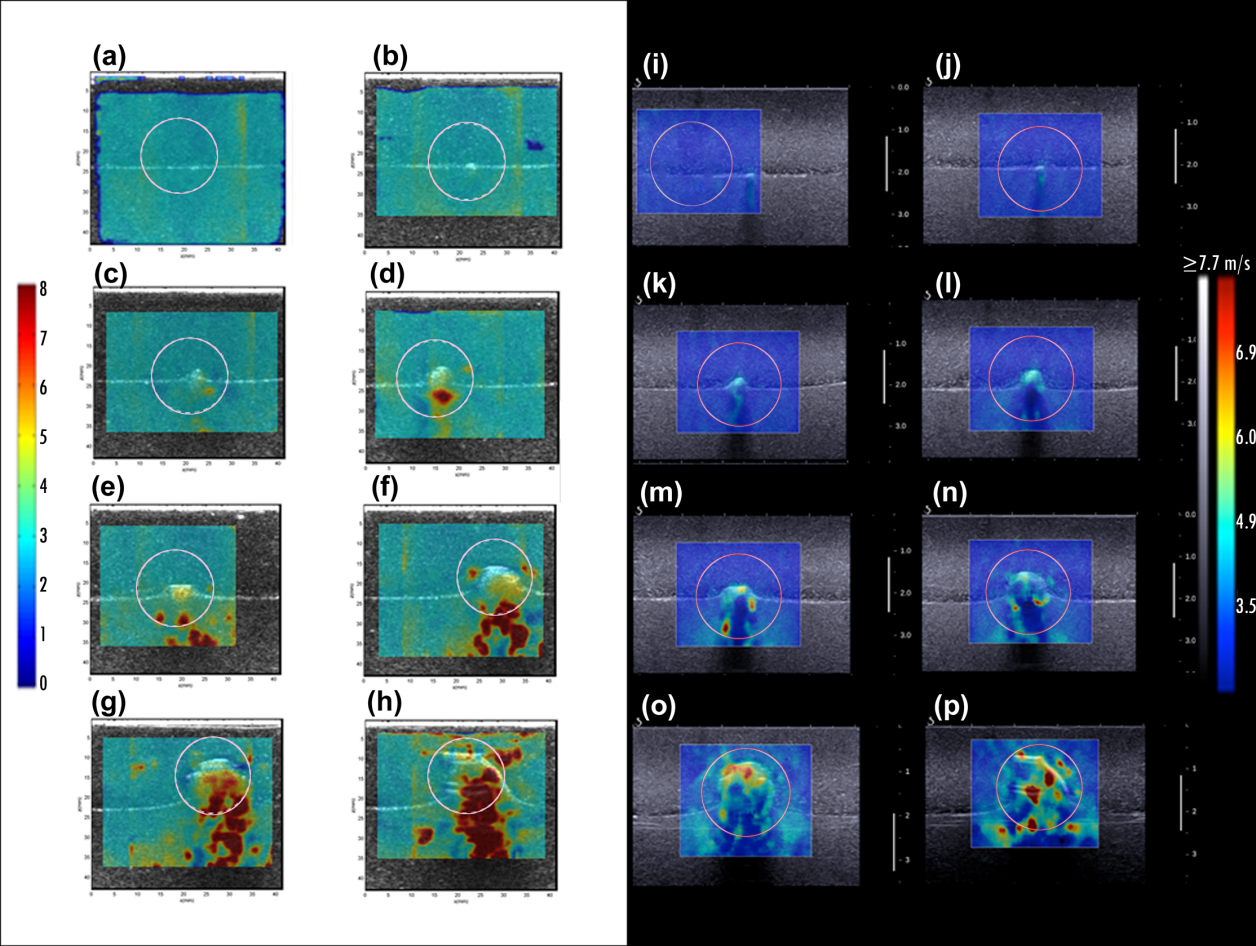
Experiment 1 results—CUSE (a-h) and SSI (i-p) shear wave speed maps. (a, i) Gelatin background (b, j) 2mm kidney stone (c, k) 3mm kidney stone (d, l) 5mm kidney stone (e, m) 6mm kidney stone (f, n) 9mm kidney stone (g, o) 11mm kidney stone (h, p) 15mm kidney stone.

Imaging of the phantom with seven kidney stone inclusions of various sizes was used to investigate the effect of single coarse calcifications on CUSE and SSI imaging. To quantify these effects, a fixed circular ROI with a diameter of 2cm was drawn around the calcification simulating an imaginary benign mass with the same elasticity value as the background (Fig 6).

During this experiment an apparent increase of the shear wave speed was observed mainly on the calcification, but when the stone size was over 6mm (Fig 6e–6h and 6m–6p) some high stiffness regions started to form below the kidney stone. These regions became more prominent for the larger stones in both CUSE and SSI imaging. We refer to the elasticity value of kidney stones as “apparent” because the real elasticity value for kidney stones is in the order of GPa.

The average of the maximum, minimum and mean elasticity from the measurements on the three acquisitions for each calcification size are shown in Table 1.The standard deviation of the mean value is the variation across the three acquisitions.

**Table 1:** Minimum, mean and maximum elasticity parameters by kidney stone size within a 2cm diameter ROI.

	CUSE			SSI		
KS size (mm)	E_min_ (kPa)	E_mean_ ± SD. (kPa)	E_max_ (kPa)	E_min_ (kPa)	E_mean_ ± SD. (kPa)	E_max_ (kPa)
0*	17.57	22.40 ± 0.90	27.54	21.9	22.70 ± 0.36	30.5
2	16.43	26.55 ± 0.13	57.06	20.9	26.95 ± 0.21	52.60
3	15.19	27.48 ± 2.44	131.97	18	27.57 ±0.32	66.20
5	9.94	32.68 ± 1.24	192.00	4.9	28.50 ± 0.14	75.70
6	21.55	33.91 ± 3.14	192.00	9.2	36.90 ± 0.28	173.10
9	16.15	41.04 ± 1.02	192.00	0.1	37.63 ± 1.12	180.00
11	16.01	59.01 ± 7.89	192.00	18.6	56.55 ± 1.91	185.40
15	21.07	73.27 ± 5.87	192.00	16.4	70.00 ± 0.99	213.50

*Background without calcifications.

**Fig 7:**
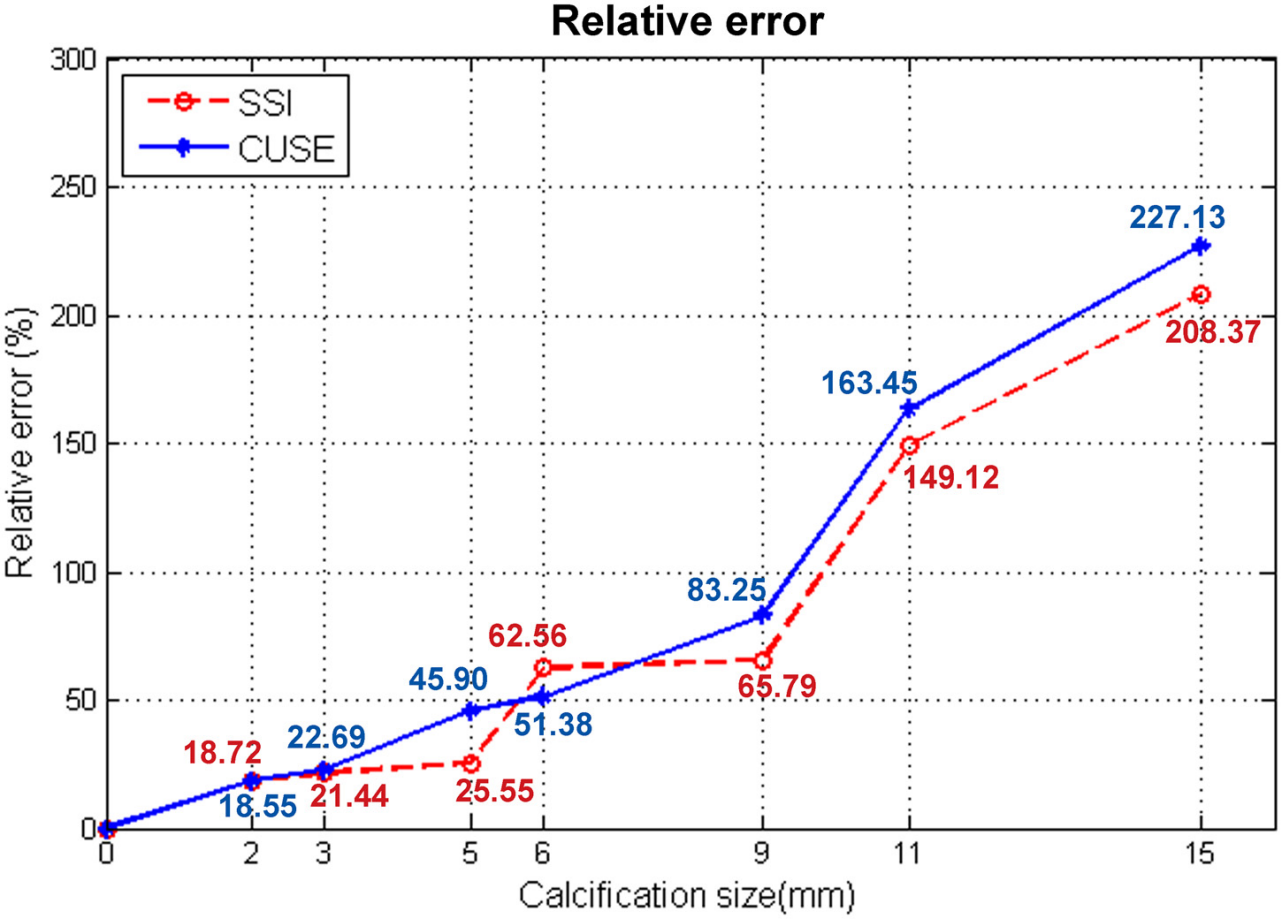
Relative error calculated from the Emean values of the 7 sizes of kidney stones and gelatin background.

The initial phantom results of the first experiment demonstrate a proportional relation between the mean and maximum elastic modulus and the diameter of the macrocalcification. E_min_ values remained within the elasticity range of the case without calcifications (background). Instead, E_mean_ values showed a significant increment particularly for the 9mm, 11mm and 15mm sizes of kidney stones. E_max_ results revealed a large increment even with the presence of the 2mm macrocalcification. We observed low variability on the three shear wave measurements obtained from calcifications smaller than 9mm (S.D. > 3.5kPa), and variations up to 7.89kPa for the larger calcifications. The apparent high speed shear waves, generated by calcifications, create an error in the elasticity measurements. In Fig 7, the relative error, calculated from Eq (2), is measured as a function of the calcification diameter for CUSE and SSI.

A good correlation can be seen between the two methods. We observed an increment of approximately 10% in the relative error per 1mm added to the macrocalcification size for inclusions between 2mm and 9mm. The relative error was above 100% for larger macrocalcifications (calcification diameter >50% of the ROI diameter).

#### Experiment 2—Study on the effect of calcifications distributions

**Fig 8:**
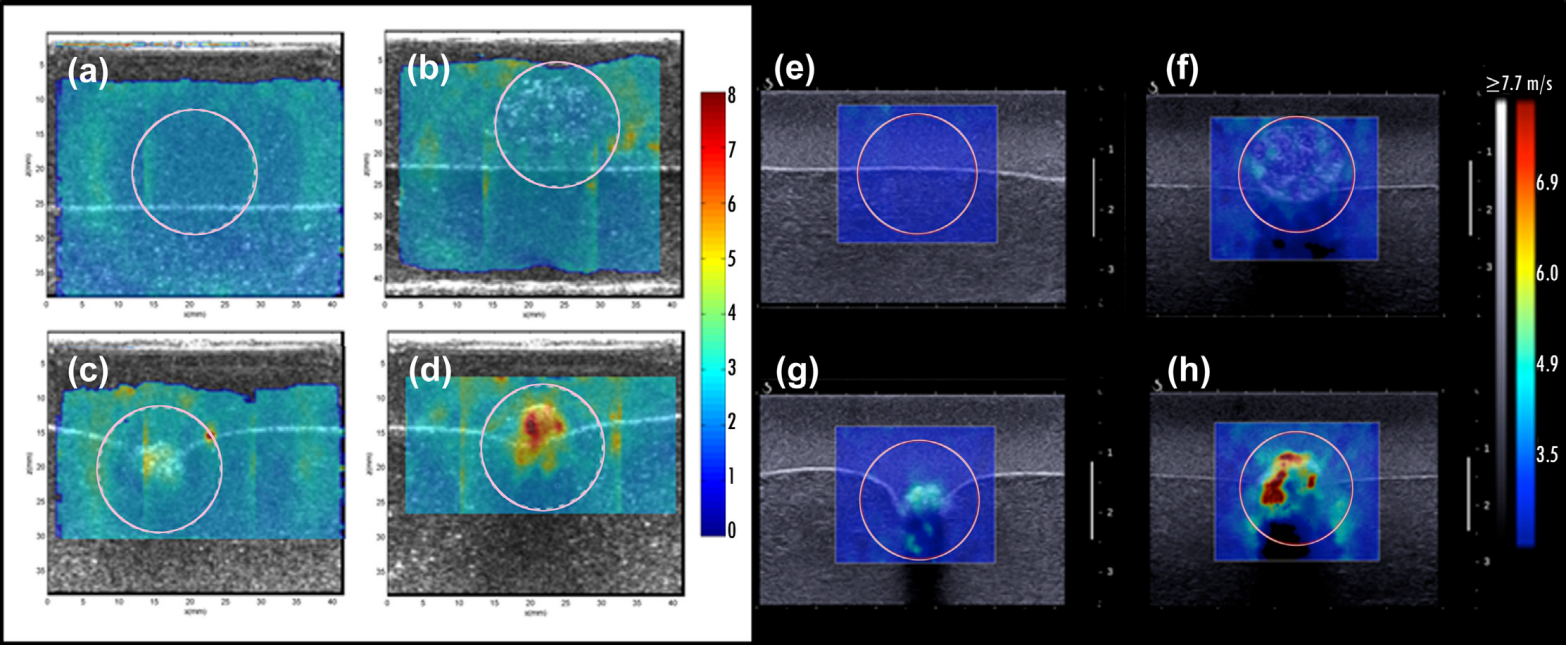
Experiment 2 results: CUSE (a-d) and SSI (e-h) shear wave speed maps. (a, e) Gelatin background (b, f) 15mm regional scattered distribution of ground kidney stone (c, g) 8mm clustered distribution of ground kidney stone (d, h) 7mm intact kidney stone.

The second experiment was assessed in a similar manner as the first phantom study. A 2cm-diameter circular ROI was drawn around the calcification area simulating an imaginary border of a mass. Fig 8 shows the resultant shear wave speed maps of CUSE and SSI techniques.

SWE images demonstrated an increment of the shear wave speed with an increase of the calcifications concentration. Table 2 summarizes the average of E_min_, E_max_ and E_mean_ values calculated from the three different acquisitions.

**Table 2:** Minimum, mean and maximum elasticity values by inclusion feature within a 2cm ROI.

	CUSE			SSI		
Kidney Stone feature	E_min_ (kPa)	E_mean_ ± SD (kPa)	E_max_ (kPa)	E_min_ (kPa)	E_mean_ ± SD (kPa)	E_max_ (kPa)
Solid	10.83	40.23 ± 0.92	192.00	0.00	46.20 ± 3.29	185.70
Cluster	13.48	36.75 ± 0.38	118.19	7.30	39.17 ± 0.49	85.10
Regional scattered	0.00	32.97 ± 2.67	86.79	10.00	29.40 ± 0.96	57.40
Background*	6.75	20.19± 1.32	24.52	11.00	20.03 ± 1.29	23.30

* Without calcifications.

The results of this experiment confirm that the distribution of microcalcifications affects the resultant elastic modulus (E_mean_ and E_max_). E_min_, similar to experiment 1, remained unbiased with the presence of calcifications.

**Fig 9:**
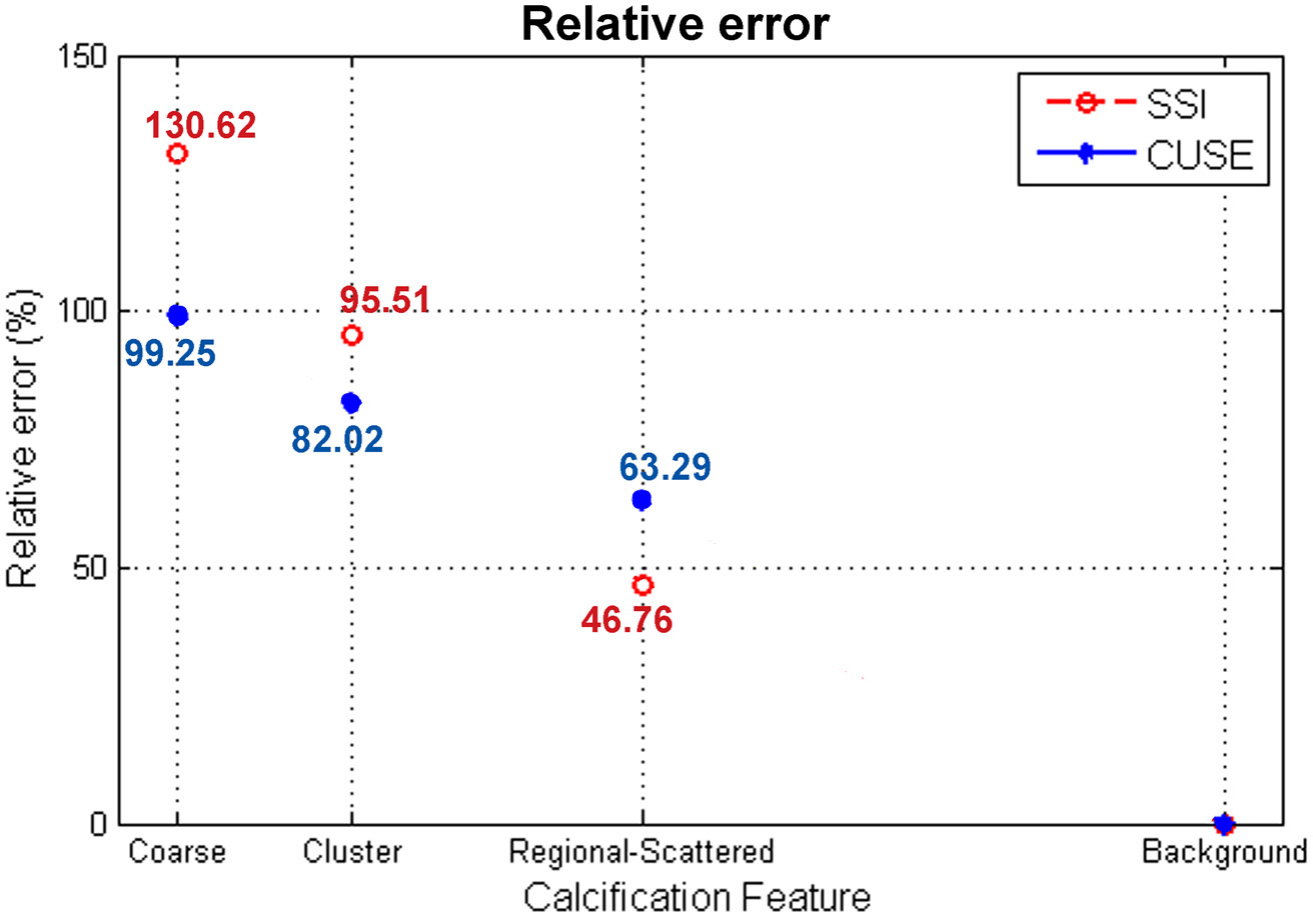
E_mean_ relative error values for a 7mm intact kidney stone (coarse), 8mm cluster distribution of ground kidney stone, 15mm regional scattered distribution of ground kidney stone, and gelatin background.

A plot of the relative error defined in Eq (2) is shown in Fig 9; a decrease of stiffness can be seen as the distribution of calcifications becomes more scattered.

#### Experiment 3—Study of the apparent increase in shear wave speed due to presence of calcifications

**Fig 10:**
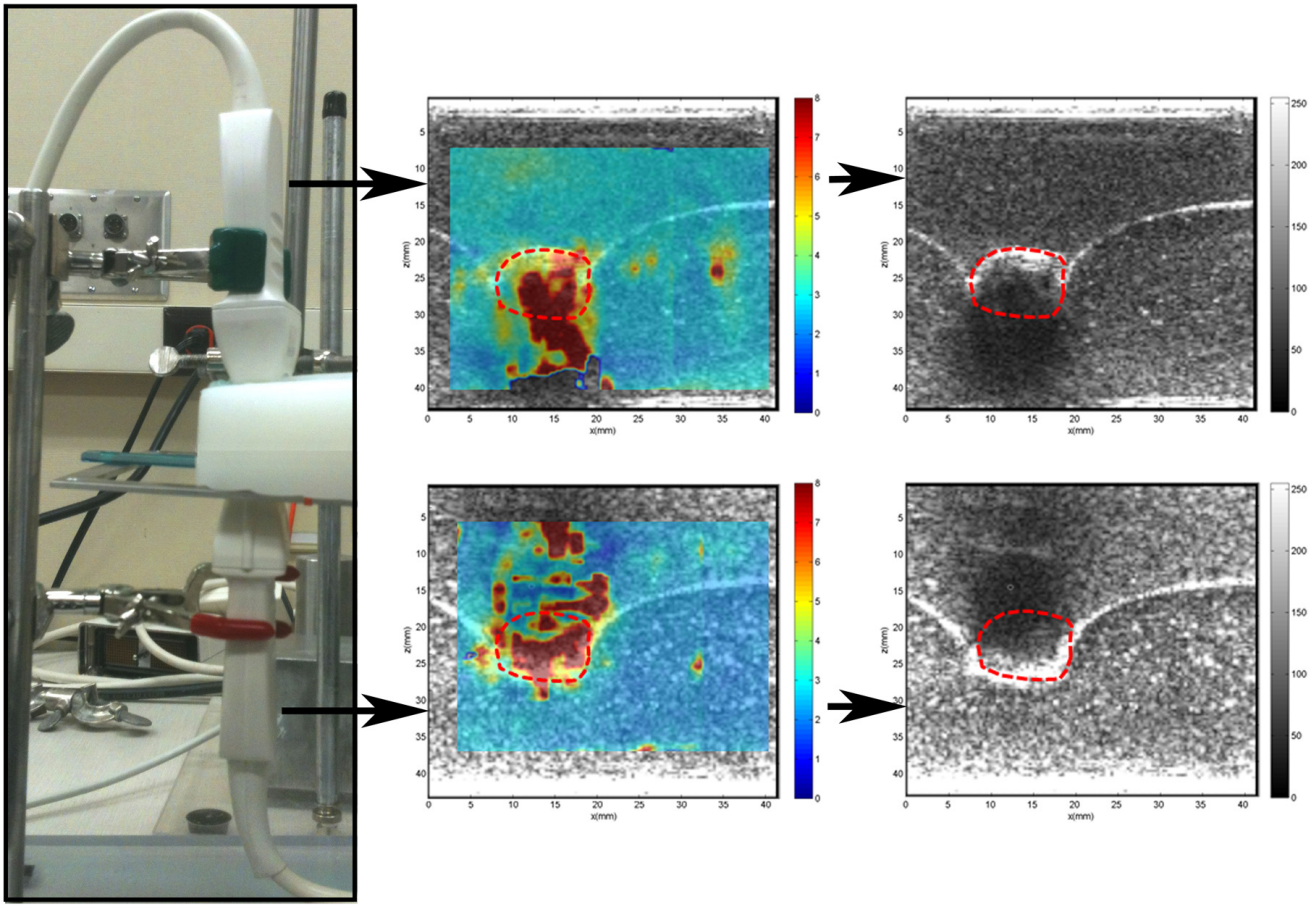
Shear wave speed maps and B-mode ultrasound of two probes aligned at opposite sides of a phantom with an 11mm kidney stone inclusion (red dashed-line).

Fig 10 shows the resultant shear wave speed maps corresponding to the transducers at the top and bottom of the phantom. It was observed that high stiffness regions were created in the shadow area directly behind the calcification (kidney stone) in both scenarios. This misinterpretation of the gelatin stiffness was considered artifact; meaning that the measurements in the calcification shadow area are not reliable.

### *In vivo* human study results

#### Case 1

**Fig 11:**
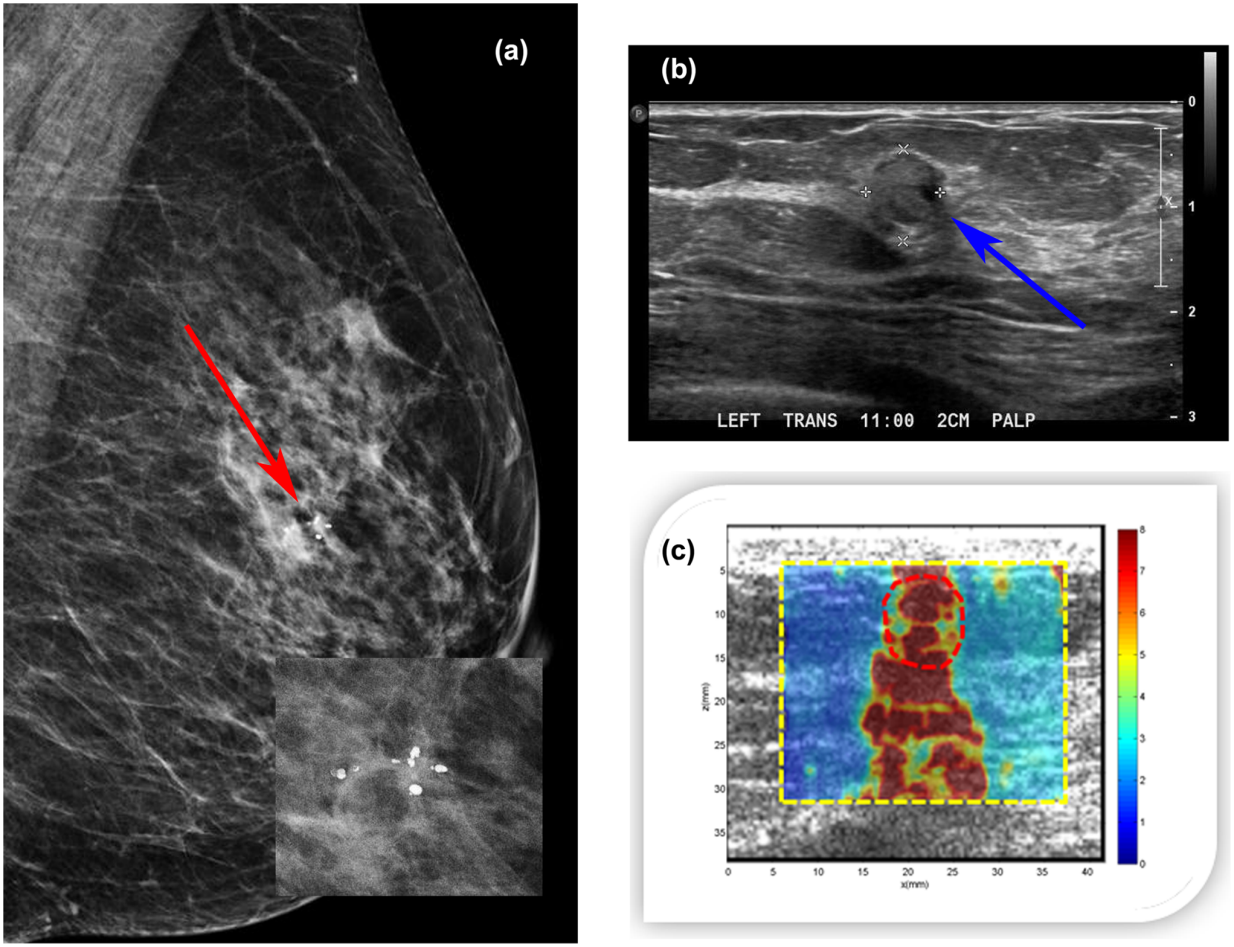
(a) Mediolateral oblique mammogram revealing a group of macrocalcifications associated with a soft tissue mass (red arrow). (b) Clinical B-mode ultrasound revealing a 6mm mass (blue arrow) with heterogeneous echogenicity. (c) CUSE image showing high stiffness along the vertical axis of the mass. The color bar indicates the speed of the shear wave.

A 47-year-old woman presented with a palpable abnormality of the left breast. Clinical mammography (Fig 11a) revealed scattered fibroglandular densities in the left breast classified as D2 and a group of calcifications within a soft tissue mass. The final assessment was BI-RADS category 4. Targeted ultrasound of the left breast demonstrated a mass measuring approximately 6 mm in greatest dimension with heterogeneous echogenicity (Fig 11b). The CUSE shear wave speed map (Fig 11c) showed high stiffness on the mass and below (in the shadow area). E_mean_ for the ROI around the mass (marked with a red dashed line) was 106.56±14.56kPa, and E_max_ was 192.00kPa. Ultrasound-guided biopsy revealed a benign organizing fat necrosis with abundant hemosiderin-laden macrophages and dystrophic calcifications in stroma.

#### Case 2

**Fig 12:**
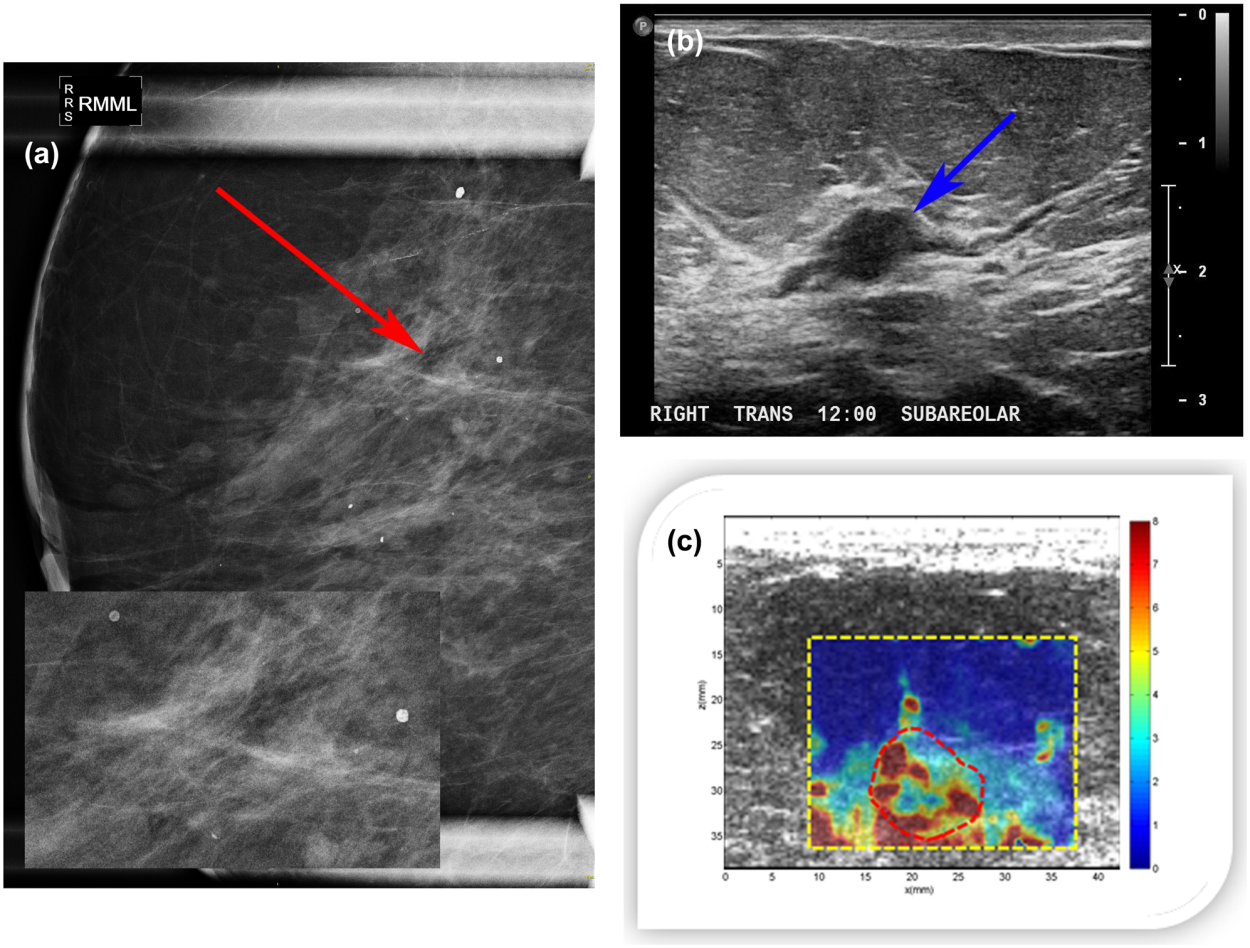
(a) Right breast magnification mediolateral mammogram showing heterogeneous dense tissue with a mix of macrocalcifications and linear microcalcifications (red arrow). (b) B-mode ultrasound of the right breast revealing a 9×6×11mm hypoechoic intraductal mass (blue arrow). (c) CUSE image showing high shear wave speed in some areas within the mass. The color bar indicates the speed of the shear wave.

A 79-year-old woman presented with new nipple inversion of the right breast. Diagnostic mammography revealed heterogeneous dense tissue in both breasts classified as D3 and area of linear calcifications in right breast (Fig 12a). Targeted ultrasound evaluation of the sub-areolar right breast demonstrated a 9×6×11mm hypoechoic intraductal mass (Fig 12b). The margins of the mass appeared indistinct in some areas. Color flow imaging demonstrated no internal vascularity. This abnormality was not seen on mammography. The final assessment was BI-RADS category 4. CUSE imaging of the right breast (Fig 12c) showed high stiffness inside the mass and surrounding area. The stiffness below the mass was due to the chest wall. E_mean_ and E_max_ within the ROI (marked around the mass with a red dashed line) were 73.21±11.29kPa and 192.00kPa, respectively. Ultrasound-guided biopsy revealed benign-focal atypical lobular hyperplasia in a background of ductal hyperplasia with calcifications present in benign ducts.

#### Case 3

**Fig 13:**
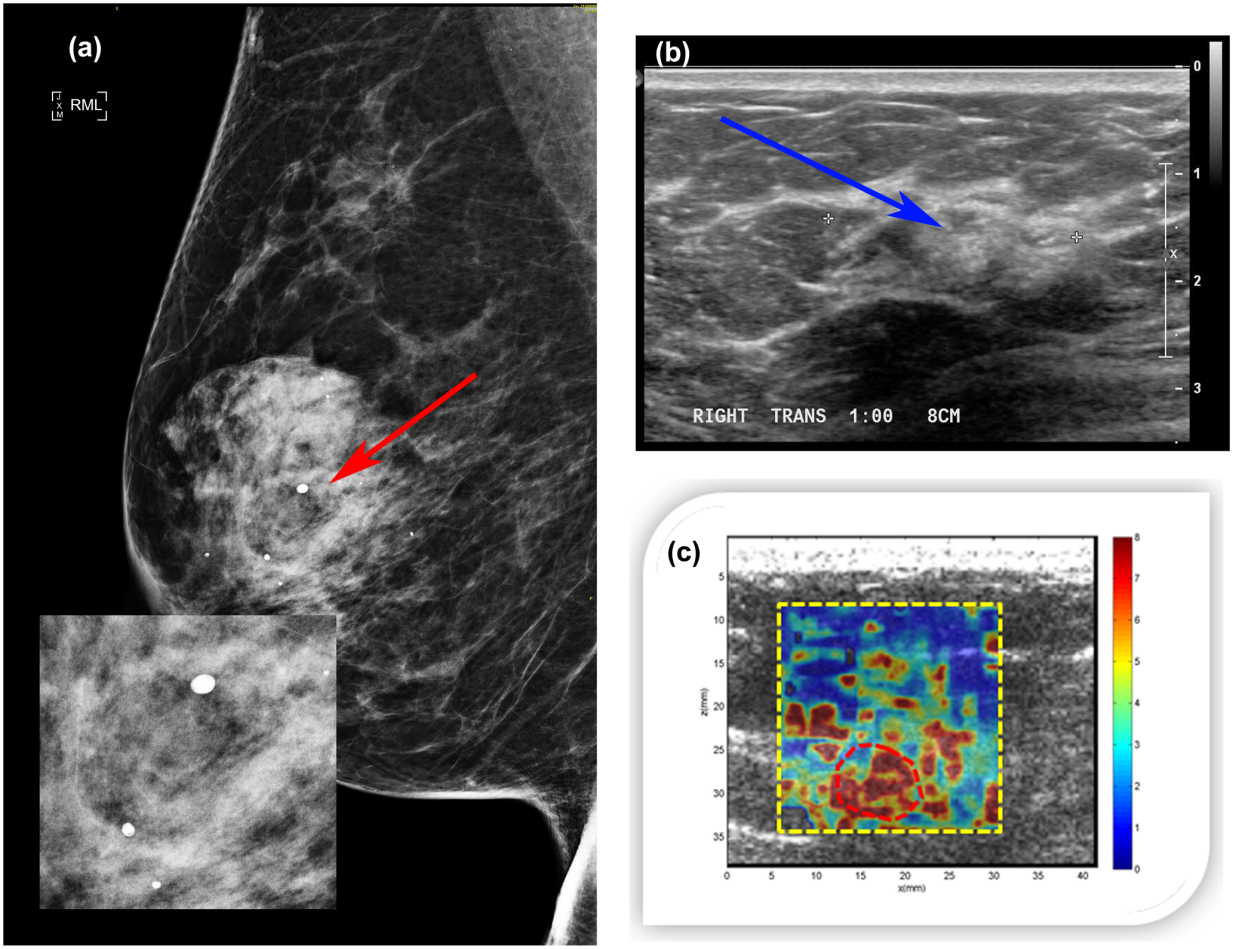
(a) Right breast mediolateral mammogram revealing a mass involving macrocalcifications (red arrow). (b) B-mode ultrasound presenting a 2.3 cm irregular area of mixed echogenicity (blue arrow). (c) CUSE image showing high stiffness on the mass; the color bar indicates the speed of the shear wave.

A 72-year-old woman presented with an abnormal breast exam of the right breast. Diagnostic mammogram revealed a 3 cm irregular asymmetry within the right breast including macrocalcifications (Fig 13a). Breast density was classified as D3. The final assessment was BI-RADS category 4. Clinical ultrasound demonstrated a 2.3cm irregular area of mixed echogenicity (Fig 13b). CUSE imaging showed high stiffness on the mass and surrounding area. Elasticity results within the ROI (marked around the mass with a red dashed line) were E_mean_ 94.08±12.7kPa and E_max_ 192.00kPa (Fig 13c). Ultrasound-guided biopsy revealed benign parenchyma with dense stromal fibrosis and calcifications in benign ducts.

## Discussion

Many studies in the differentiation of benign from malignant breast masses have used several elasticity parameters to correlate with the pathology. Some of the most commonly used in SSI studies are E_mean_ and E_max_ with cut-off values ranging from 45kPa to 80kPa for E_mean_ and from 54kPa to 94kPa for E_max_ [41, 42, 48–52]. In our initial results from the CUSE study for differentiation of benign and malignant breast lesions E_mean_ was associated to the results of pathology with a cut-off value of 83kPa [27].

In this study, highly dense clusters of microcalcifications and single macrocalcifications larger than 5mm created the appearance of high shear wave speeds above 5.77m/s, corresponding to E_max_ 100kPa. Regional scattered microcalcifications did not reach as high values but were able to increase the elastic modulus of the ROI (imaginary mass) in approximately 50% for E_mean_ and 250% for E_max_.

Berg et. al [40] in a multinational study using a prototype of the Aixplorer scanner equipped with a L15-4 transducer reported an increase of E_max_ 9kPa for masses associated with calcifications. Our phantom studies confirm that calcifications can induce the appearance of high stiffness. SSI phantom results showed an increase in E_max_ of 26kPa for our smallest macrocalcification (2mm-in diameter) and 31kPa for the inclusion with regional scattered distribution of microcalcifications (15mm-in diameter). The cause for the discrepancy of E_max_ between the two studies could be due to the size of the calcifications, degree of concentration of microcalcifications within the ROI, and/or the elasticity value of the background (mass).

The increase of E_mean_ due to presence of calcifications is dependent on the size of the ROI, and the size of the ROI should be considered when comparing the results with others. However, the only study in this area was done by Berg et. al [40], where they studied the effect of calcifications on E_max_. To the best of our knowledge, no studies have been done on the effect of calcification on E_mean_ values to compare with our results.

The relative error measured the percentage of changes in the mean elasticity values due to the presence of calcifications. We observed an increase of the relative error with an increase in the concentration or calcification size. More than a 100% increase in the E_max_ relative error is expected with the presence of macrocalcifications larger than 2mm of diameter. The degree of concentration of microcalcifications is another variable that affects shear wave elasticity measurements. Dispersedly distributed microcalcifications extended throughout the inclusion have less effect than concentrated clusters of microcalcifications located in a fraction of the inclusion. Similar to macrocalcifications, E_mean_ measurements of clustered microcalcifications depend on the ROI size. In the phantom study, 40% of the ROI area contained the cluster of microcalcifications resulting in ~90% E_mean_ relative error.

Although we used two systems with different probes and different excitation methods, our results showed good correlation between them. However, a comparison between these two systems is beyond the scope of this paper.

The third phantom experiment, where two transducers were used, demonstrates that the high stiffness error below macrocalcifications results from low signal-to-noise ratio of the B-mode in the shadow area. This apparent stiffness is considered artifact; meaning that the measurements in calcification shadow area are not reliable.

An additional factor that has a direct effect when it comes to the differentiation of benign and malignant masses is the cut-off value for elasticity measurements. Low cut-off values are more sensitive to the presence of calcifications and could increase the number of false positives.

Our phantoms results demonstrated to be reproducible (low standard deviation) across three different acquisitions. More variations on the mean elasticity are observed in the *in vivo* studies where the background is heterogeneous.

The *in vivo* human study results showed that different types of calcifications affect the elasticity at different degrees. In case 1, the mass with the group of large macrocalcifications showed the highest stiffness of the three cases, followed by the mass involving microcalcifications in benign ducts with a large macrocalcification (case 3), and finally the mass with a small macrocalcification and microcalcifications in benign ducts showed the lowest stiffness (case 2). Two of the three masses would be misclassified considering the reported CUSE cut-off value of E_mean_ 83kPa [27]. Our *in vivo* findings are in agreement with those from Berg et. al [40], showing that the presence of calcifications in a mass is correlated with increasing stiffness.

## Conclusions

The results from our study demonstrate that the presence of clustered and large or coarse calcifications in benign breast masses can induce the appearance of high stiffness regions when they are evaluated by shear wave elastography methods. Consequently, such benign masses can be misdiagnosed as malignant. Although we have not fully investigated the sources of such errors, our experimental results with two probes looking from two sides of a tissue mimicking phantom and the fact that the error for two SWE systems have similar patterns suggest that it is mostly due to the inaccurate tracking of shear waves.

Studies with larger patient populations are needed to further quantify the elasticity elevations in benign masses with various types of calcification.
