# Correction: Effect of Calcifications on Breast Ultrasound Shear Wave Elastography: An Investigational Study

**DOI:** 10.1371/journal.pone.0147462

**Published:** 2016-01-14

**Authors:** Adriana Gregory, Mohammad Mehrmohammadi, Max Denis, Mahdi Bayat, Daniela L. Stan, Mostafa Fatemi, Azra Alizad

The following information is missing from the Funding section: This work is supported by grants R01CA148994, R01CA174723, R01 EB017213, and R01CA168575 from the National Institutes of Health-National Cancer Institute. The funders had no role in study design, data collection and analysis, decision to publish, or preparation of the manuscript.
